# The expression and function of human CD300 receptors on blood circulating mononuclear cells are distinct in neonates and adults

**DOI:** 10.1038/srep32693

**Published:** 2016-09-06

**Authors:** Olatz Zenarruzabeitia, Joana Vitallé, Susana García-Obregón, Itziar Astigarraga, Cristina Eguizabal, Silvia Santos, Venkateswara R. Simhadri, Francisco Borrego

**Affiliations:** 1 Immunopathology Group, BioCruces Health Research Institute, Barakaldo, Spain; 2 Pediatric Oncology Group, BioCruces Health Research Institute, Barakaldo, Spain; 3 Pediatrics Service, Cruces University Hospital, Barakaldo, Spain; 4 Department of Pediatrics, Faculty of Medicine and Dentistry. University of the Basque Country, Leioa, Spain; 5 Cell Therapy and Stem Cell Group, Basque Center for Transfusion and Human Tissues, Galdakao, Spain; 6 Division of Biotechnology Review and Research-I, Office of Biotechnology Products Review and Research, CDER, Food and Drug Administration, USA; 7 Ikerbasque, Basque Foundation for Science, Bilbao, Spain

## Abstract

Neonates are more susceptible to infections than adults. This susceptibility is thought to reflect neonates’ qualitative and quantitative defects in the adaptive and innate immune responses. Differential expression of cell surface receptors may result in altered thresholds of neonatal immune cell activation. We determined whether the expression and function of the lipid-binding CD300 family of receptors are different on neonatal immune cells compared to adult immune cells. A multiparametric flow cytometry analysis was performed to determine the expression of CD300 receptors on adult peripheral blood mononuclear cells and neonatal cord blood mononuclear cells. The expression of the CD300a inhibitory receptor was significantly reduced on cells from the newborn adaptive immune system, and neonatal antigen presenting cells exhibited a different CD300 receptors expression pattern. We also found differential LPS-mediated regulation of CD300 receptors expression on adult monocytes compared to cord blood monocytes, and that CD300c and CD300e-mediated activation was quantitatively different in neonatal monocytes. This is the first complete study examining the expression of CD300 receptors on human neonatal immune cells compared with adult immune cells. Significant differences in the expression and function of CD300 receptors may help to explain the peculiarities and distinctness of the neonatal immune responses.

It is well known that neonates are more susceptible to infectious agents than adults^1^,^2^,^3^. This increased susceptibility to infection is, at least in part, due to immaturity and naiveté of their immune system, affecting both the innate and adaptive immune responses^4^,^5^,^6^. For instance, it has been described that neonatal antigen presenting cells (APCs) are low in numbers, express lower major histocompatibility complex class II molecules (MHC-II), CD80 and CD86, differently respond to toll-like receptor (TLR) agonists, have a decreased ability to generate T helper 1 (Th1) responses and a marked decrease in the production of pro-inflammatory cytokines such as type 1 interferon (IFN) or tumour necrosis factor alpha (TNF-α)^5^,^7^,^8^,^9^,^10^,^11^,^12^,^13^. The lymphoid compartment in the newborn also exhibits qualitative and quantitative differences^5^,^14^. For example, it has been described that neonatal natural killer (NK) cells display an increased expression of the inhibitory receptor CD94/NKG2A and less cytotoxic activity than adult NK cells^15^,^16^,^17^. These differences in the newborn immune system could be crucial for protection during the transition from a sterile environment, the womb, to the outside world that is saturated with antigens, and thus avoid exuberant immune responses with the consequent danger that this would entail. In spite of the numerous findings already described, still we have an incomplete picture of the differences between neonatal and adult immune systems.

In order to preserve the identity and integrity of the host and at the same time being effective against insults, a balance between stimulating and inhibitory signals is required to adjust the activation status of the immune system. Among several other mechanisms that achieve this task, the balance is accomplished by signals that originate from cell surface receptors with activating and inhibitory capabilities^18^,^19^,^20^. The human CD300 family consists of 8 receptors encoded in chromosome 17 and they are expressed in both myeloid and lymphoid lineages, except CD300g that is expressed on endothelial cells. The CD300 molecules are type I transmembrane proteins with a single immunoglobulin (Ig)V-like extracellular domain. CD300a and CD300f receptors have a long cytoplasmic tail with immunoreceptor tyrosine-based inhibitory motifs (ITIMs) which are required for the inhibitory signalling; while other members (CD300b, CD300c, CD300d, CD300e and CD300h), have a short cytoplasmic tail and a charged amino acid residue that allows their association with immunoreceptor tyrosine-based activating motifs (ITAM)-bearing adaptors which transduce activation signals. The biological and clinical significance of CD300 molecules and their participation in the pathogenesis of numerous diseases such as allergies, psoriasis, leukaemia, sepsis, etc. have been documented over the last years^21^,^22^,^23^,^24^.

The knowledge about the expression and signalling-mediated abilities of the CD300 receptors in human newborn immune cells is nearly non-existent. Here, we have performed a comprehensive comparative analysis of the expression of this family of receptors on adult peripheral blood mononuclear cells (PBMCs) versus neonatal cord blood mononuclear cells (CBMCs). In addition, we have studied the regulation of the expression of certain CD300 members on monocytes and their functional capabilities. Our results reveal significant differences in the expression and function of these receptors that may help to explain the idiosyncrasies of the neonatal immune system.

## Results

### CD300 molecules are differentially expressed on peripheral blood adult immune system cell populations and subpopulations

Currently, a methodical study describing CD300 receptors expression in human mononuclear cells is absent. Therefore, we systematically analysed the expression pattern of CD300a, CD300c, CD300e and CD300f on adult PBMCs. We have chosen these receptor family members because they are expressed on the cell surface of immune cells and there are available mAbs for their detection by flow cytometric analysis. Even though there are commercially available specific anti-CD300c, anti-CD300e and anti-CD300f mAbs, to our knowledge, there is not a specific anti-CD300a fluorochrome conjugated mAb. In this study, we have used the clone E59.126, which recognizes both CD300a and CD300c, side by side with the specific anti-CD300c clone TX45 with the objective to distinguish the cell surface expression of the two receptors^25^.

**Figure 1:**
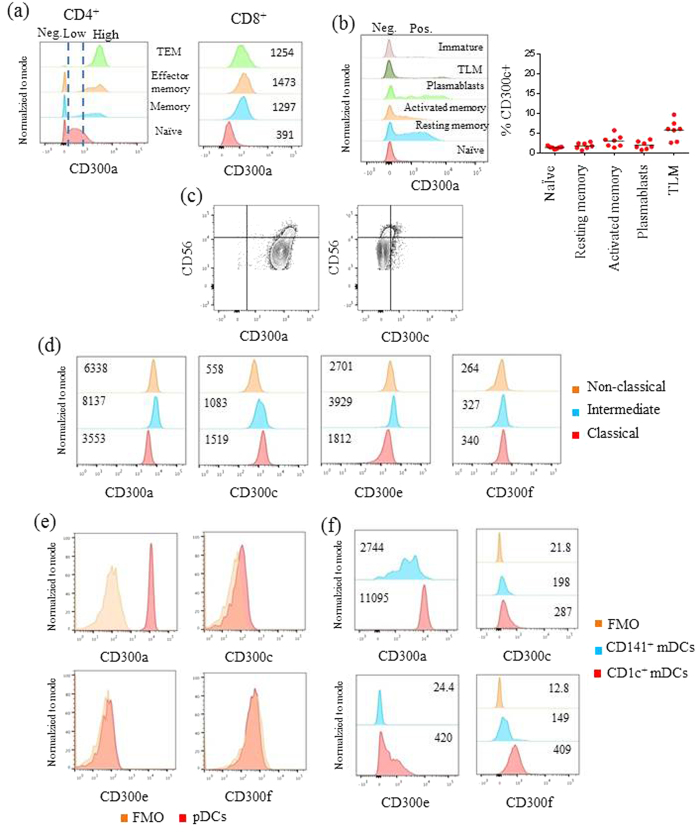
CD300 molecules expression on adult peripheral blood mononuclear cells. (**a**) The expression of CD300a was assessed on CD4^+^ and CD8^+^ naïve (red), memory (blue), effector/memory (orange) and TEM (green) T cells. Numbers represent the median fluorescence intensity (MFI) of CD300a expression. **(b)** The expression of CD300a was assessed on naïve (red), resting memory (blue), activated memory (orange), plasmablasts (green), TLM (dark green) and immature (pink) B cells. The dot graph represents the percentage of CD300c^+^ cells in each B cell subset. **(c)** Contour plots representing the expression of CD300a and CD300c on CD56^dim^ and CD56^bright^ NK cell subsets. **(d)** The expression of CD300a, CD300c, CD300e and CD300f were assessed on classical (red), intermediate (blue) and non-classical (orange) monocytes. Numbers represent the MFI of CD300 molecules expression. **(e)** The expression of CD300a, CD300c, CD300e and CD300f were assessed on pDCs. Red histograms represent the binding of anti-CD300 mAbs, and the orange histograms represent the FMO control. **(f)** The expression of CD300a, CD300c, CD300e and CD300f were assessed on CD1c^+^ (red) and CD141^+^ (blue) mDCs. The red and blue histograms represent the binding of anti-CD300 mAbs; and the orange histograms represent the FMO control. For the detection of the CD300 molecules, the following mAbs were used: clone E59.126 for CD300a (**c**), clone TX45 for CD300c, clone UP-H2 for CD300e and clone UP-D2 for CD300f. Histograms and contour plots are representative of data obtained from 7–9 healthy donors.

It is known that human T lymphocytes from healthy adult’s peripheral blood express CD300a^26^,^27^,^28^,^29^. On CD4^+^ T cells, the expression of CD300a is associated with Th1 cells that are more polyfunctional, and on CD8^+^ T cells, CD300a expression is mostly associated with effector functions^26^,^28^. However, these studies were performed with the clone E59.126 without excluding the possibility that T cells also express CD300c. Here, by using the specific anti-CD300c clone TX45, we have determined that human T cells do not express CD300c (data not shown), therefore indicating that clone E59.126 is only detecting CD300a on T cells. Next, we have analysed the surface expression of CD300a in naïve (CD45RA^+^CD27^+^), memory (CD45RA^−^CD27^+^), effector/memory (CD45RA^−^CD27^−^), and terminal effector/memory (TEM) (CD45RA^+^CD27^−^) T cells (Fig. 1a and Supplementary Figure 1a). CD4^+^ naïve T cells could be divided into two groups based upon the expression of CD300a, the CD300a^neg^ and the CD300a^low^ subsets. On the other hand, in CD4^+^ memory, effector/memory and TEM cells two subsets were also distinguished, i.e. CD300a^neg^ and CD300a^high^. It is important to note that the majority (>90%) of TEM CD4^+^ T cells were CD300a^high^. On CD8^+^ T cells, all subpopulations expressed uniform levels of CD300a, but the expression in memory, effector/memory and TEM subsets was 5–6 folds higher when compared to the expression levels of CD8^+^ naïve T cells (Fig. 1a).

In order to identify mature and immature circulating human CD19^+^ B cell subsets, we used a staining strategy based on the expression of CD10, CD20, CD21 and CD27 (Supplementary Figure 1b). Immature B cells are characterized by the expression of CD10, while mature B cells are CD10^−^. Five mature B cell subpopulations are distinguished: naïve cells (CD21^+^CD27^−^), resting memory cells (CD21^+^CD27^+^), activated memory cells (CD21^−^CD27^+^CD20^+^), plasmablasts (CD21^−^CD27^+^CD20^−^) and tissue-like memory (TLM) B cells (CD21^−^CD27^−^). We observed that naïve B cells were mostly negative for CD300a expression, whereas the rest of B cell subsets could be divided into CD300a^+^ and CD300a^−^ populations (Fig. 1b). The lack of expression of CD300c on B cells as measured by the binding of specific anti-CD300c clone TX45, except for ≤5% of the TLM and activated memory B cells, indicate that clone E59.126 is mostly detecting CD300a on the cell surface of B cells. When we analysed CD10^+^ immature circulating B cells, we observed that there is a small subset of CD300a^+^ cells (Fig. 1b).

Human NK cells are phenotypically characterized by the expression of CD56 and the lack of CD3 on their cell surface. Examining the surface density of CD56 expression, NK cells are divided into two distinct subsets, CD56^bright^ and CD56^dim^ (Supplementary Figure 1c). In the periphery, approximately 90% of human NK cells are CD56^dim^ expressing high levels of CD16 (FcγRIII) and are predominantly cytotoxic in function. In contrast, only 5–10% of NK cells are CD56^bright^ and CD16^dim/neg^ with a predilection for secreting pro-inflammatory cytokines^30^. Focusing on the cell surface expression of CD300 molecules, both CD56^dim^ and CD56^bright^ subsets expressed CD300a, with the latter subset having a tendency to express higher levels. On the other hand, only CD56^bright^ NK cells expressed very low or negative levels of CD300c, while the CD56^dim^ subset did not show any expression of this receptor (Fig. 1c), indicating that clone E59.126 only is detecting CD300a. NK cells, like the other lymphocyte populations analysed, did not express CD300e neither CD300f (data not shown).

Three subsets of monocytes are distinguished based on the expression of CD14 and CD16, namely classical monocytes (CD14^++^CD16^−^), intermediate monocytes (CD14^++^CD16^+^) and nonclassical monocytes (CD14^+^CD16^+^) (Supplementary Figure 1d)^31^. Previously, it has been published that monocytes from adult peripheral blood express CD300a, c, e and f 25,32,33,34, although the expression of these receptors was not analysed in each monocyte subset. Therefore, we determined the expression of CD300 receptors and found that intermediate and nonclassical monocytes exhibited higher cell surface expression levels of CD300a and CD300e than classical monocytes, while the latter showed the highest expression of CD300c. In relation to CD300f expression, the three monocytes subpopulations showed similar levels, with the nonclassical monocytes displaying a tendency to express less CD300f (Fig. 1d).

According to Ziegler-Heitbrock *et al.*, there are three types of human dendritic cells (DC) in blood, that is, plasmacytoid DC (pDC) and two types of myeloid DCs (mDCs) (Supplementary Figure 1e)^31^. DCs are identified for being lineage marker negative cells and HLA-DR positive, and in the case of pDCs they are specifically recognized by the expression of CD303 (Supplementary Figure 1f). Cell surface expression analysis of CD300 receptor family members showed that pDCs express CD300a. They exhibited none or negligible expression of other CD300 molecules (Fig. 1e). As mentioned before, two types of blood mDCs are distinguished, one subset expresses the marker CD1c and the other is identified by the expression of CD141. The CD1c^+^ mDCs, which are the vast majority, expressed the four CD300 receptor family members analysed in this study, including the expression of very high levels of the CD300a inhibitory receptor (Fig. 1f). On the other hand, the expression levels of CD300 molecules were significantly lower in the CD141^+^ mDCs compared to the CD1c^+^ mDCs (Fig. 1f).

### The expression pattern of CD300 surface receptors is different in neonatal and adult mononuclear cells

Next, we were interested in determining if a different expression pattern of CD300 surface molecules on neonatal immune cells would help to explain the differences between the newborn and adult immune responses. Therefore, we decided to systematically analyse the expression of CD300 receptors on CBMCs in comparison with adult PBMCs.

**Figure 2:**
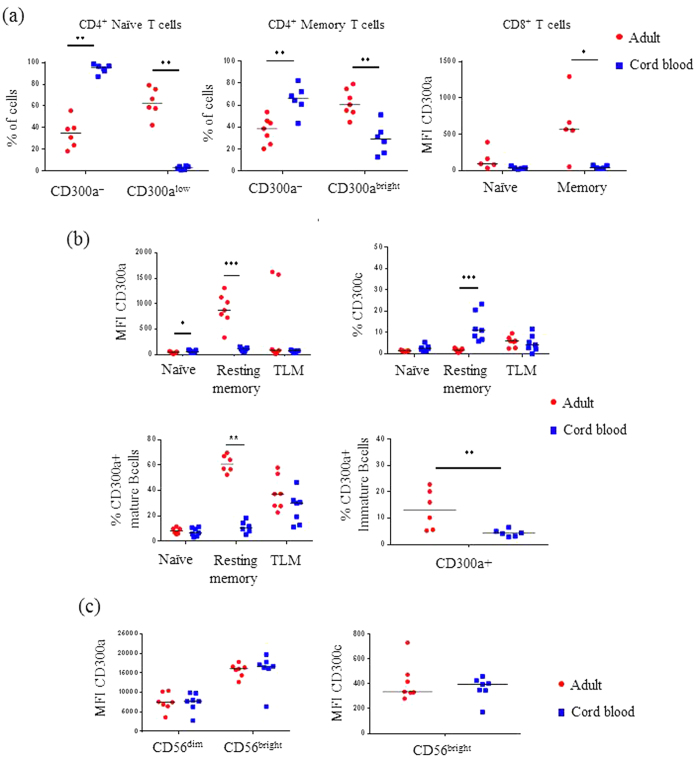
The expression pattern of CD300 surface receptors on lymphocytes is different in neonates from adults. **(a)** Dot graphs representing the percentage of CD300a^−^ and CD300^low^ cells in CD4^+^ naïve cells (left), the percentage of CD300a^−^ and CD300a^bright^ on CD4^+^ memory T cells (middle), and the MFI of CD300a expression on CD8^+^ naïve and memory T cells (right). **(b)** Dot graphs showing the MFI (upper left) of CD300a expression and the percentage (lower left) of CD300a^+^ cells in naïve, resting memory and TLM mature B cell subsets, the percentage of CD300c^+^ cells in naïve, resting memory and TLM mature B cell subsets (upper right), and the percentage of CD300a^+^ immature B cells (lower right). **(c)** Dot graphs representing the MFI of CD300a (left) and CD300c (right) expression on CD56^dim^ and CD56^bright^ NK cells is represented. Each dot represents a different donor, and the medians are represented. *p < 0.05, **p < 0.01, ***p < 0.001.

We found that almost all CD4^+^ and CD8^+^ cord blood T lymphocytes were naïve, 97% and 90% respectively, while the rest of T cells had a memory phenotype. We did not find significant amounts of memory/effector cells or TEM cells in cord blood (Supplementary Figure 2a). The vast majority (>95%) of the naïve CD4^+^ T cells from cord blood were negative for CD300a expression, while >50% of adult circulating naïve CD4^+^ T cells expressed CD300a at low levels (Fig. 2a). On the other hand, although we observed a fraction of cord blood memory CD4^+^ T cells that were positive for CD300a, the frequency of CD300a^+^ of neonatal memory CD4^+^ T cells was significantly lower when compared with adult memory CD4^+^ T cells (28.9 ± 5.7% of CD300a^+^ cells in CBMCs vs. 62.0 ± 4.7 of CD300a^+^ in adult PBMCs) (Fig. 2a). Lastly, CD300a expression was absent or very low on both naïve and memory CD8^+^ T cell from cord blood (Fig. 2a).

As in T cells, naïve B lymphocytes represented the majority of mature B cells from cord blood, while there is a small subset (<5%) of resting memory and TLM B cells. Activated memory B cells and plasmablats are almost absent from cord blood, and the frequency of CD10^+^ immature B cells was significantly higher in cord blood than in adult peripheral blood (Supplementary Figure 2b). As in adults, naïve B cells from neonates express none or very low levels of CD300a, while the expression of CD300a in memory B cells from cord blood was significantly lower than in the adult (Fig. 2b). Very interestingly, the frequency of CD300c^+^ resting memory B cells was significantly higher in CBMCs than in adult PBMCs (Fig. 2b). Finally, CD10^+^ immature B cells from cord blood exhibited significantly lower frequencies of CD300a^+^ cells than CD10^+^ immature B cells from adult blood (Fig. 2b).

Regarding NK cells, we did not observe any significant difference between the frequency of CD56^dim^ and CD56^bright^ NK cells from cord blood and adult blood (Supplementary Figure 2c). Similarly, the cell surface expression of CD300a and CD300c was very similar on NK cells from both neonates and adults (Fig. 2c). Cord blood NK cells, T cells and B cells did not express CD300e neither CD300f (data not shown).

**Figure 3:**
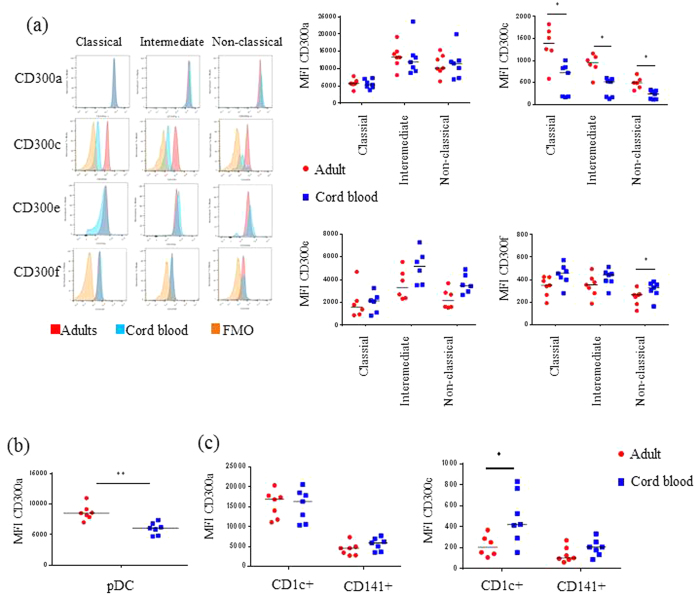
The expression pattern of CD300 surface receptors on myeloid cells is different in neonates from adults. **(a)** The expression of CD300 molecules was assessed on monocyte subsets. On the left, representative histograms are shown. On the right, dot graphs representing the MFI of CD300a, CD300c, CD300e and CD300f expression in classical, intermediate and nonclassical monocytes are shown. **(b)** Dot graphs showing the MFI of CD300a expression on pDCs. **(c)** Dot graphs representing the MFI of CD300a and CD300c in CD1c^+^ and CD141^+^ mDCs are shown. Each dot represents a different donor, and the medians are represented. *p < 0.05, ** p < 0.01.

The neonatal monocyte population is thought to be immature, but not much is known regarding the different monocyte subsets^5^,^14^. We examined the frequencies of monocyte subsets as well as the cell surface expression of CD300 molecules. We observed that the percentages of classical monocytes are slightly higher, although not statistically significant, in CBMCs than in adult PBMCs (95.7 ± 3.8% of cord blood monocytes vs. 91.7 ± 3.2% of monocytes in adults) and the percentage of intermediate monocytes were similar in both samples. However, we observed that cord blood exhibited significantly lower frequencies of nonclassical monocytes than adults (Supplementary Figure 2d). Analysis of cell surface expression of CD300 molecules on monocytes from cord blood showed that the expression of CD300c was significantly lower on all monocytes subsets from neonates compared with adult monocytes (Fig. 3a). On the other hand, we did not find differences in the expression of CD300a between adult and neonate monocytes. Intermediate and nonclassical monocytes from cord blood have a tendency to express higher levels of CD300e compared with intermediate and nonclassical monocytes from adult blood, and regarding CD300f expression we did not find any significant differences between adult and cord blood monocytes (Fig. 3a).

DCs were found in lower frequencies in cord blood than in adult peripheral blood (Supplementary Figure 2e). Like in adult PBMCs, pDCs in CBMCs only expressed CD300a, but they showed significantly lower levels than adult peripheral blood pDCs (CD300a MFI: 5979 ± 368 in CBMC vs. 8619 ± 466 in adult PBMC) (Fig. 3b). Regarding mDCs, we only observed differences in the expression levels of CD300c on the CD1^+^ mDC subset, which were higher in neonates than in adults (CD300c MFI: 486.3 ± 92.3 in neonates vs. 218.7 ± 41.1 in adults) (Fig. 3c). We did not find differences on CD300a expression between adult and neonate mDCs. CD300e and CD300f expression were similar on neonates and adults mDCs (data not shown).

Altogether, our results show that the expression of CD300 molecules is different between CBMCs and adult PBMCs. Cells from the neonatal adaptive immune system significantly expressed lower levels of the CD300a inhibitory receptor compared with cells from the adult immune system. CD300 receptors expression on monocytes from cord blood also exhibited significant differences when compared with adult monocytes. Finally, the expression levels of the CD300c activating receptor differed between adult PBMCs and CBMCs depending on the cell type, while we observed few differences on the expression of CD300e and CD300f receptors between adult and neonatal monocytes and DCs.

### LPS-mediated regulation of CD300 molecules on monocytes differs in adults and neonates

**Figure 4:**
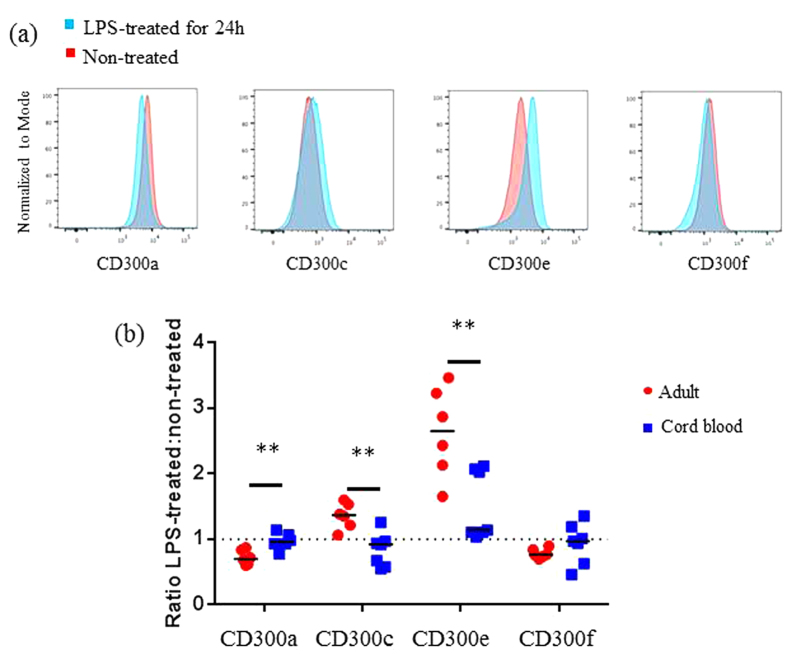
LPS-mediated regulation of CD300 molecules on neonatal and adult monocytes. Monocytes were either left untreated or treated with 10 ng/ml of LPS for 24 h. Then, cells were harvested and checked for CD300a, CD300c, CD300e and CD300f cell surface expression. **(a)** Representative histograms showing CD300 receptors modulation in adult monocytes in response to LPS. The red histograms represent the untreated condition and the blue histograms represent the LPS-treated condition. Results are illustrative of data from 7 adult healthy donors. **(b)** Dot plot graph showing CD300 receptors modulation on monocytes from adults and newborns after LPS treatment. Data are represented as the ratio of MFI of each CD300 molecule between LPS-treated and untreated cells. The horizontal dotted line (value 1) represents no change on CD300 molecules expression after LPS treatment. Each dot represents a different donor, and the medians are represented. **p < 0.01.

Next, we were interested in understanding how CD300 receptors expression is regulated on neonatal immune cells. We chose to work with monocytes because they express the four CD300 molecules tested in this study. Also, it is known that the TLR4 ligand LPS modulates the expression of CD300c on monocytes^25^. Therefore, we investigated CD300 receptors expression on adult monocytes after 24 h of LPS treatment and we observed a significant increase on CD300c and CD300e expression when compared with untreated monocytes, while the expression of CD300a and CD300f decreased (Fig. 4a). It is well accepted that cord blood monocytes are somehow less responsive to LPS^12^,^35^. For example, they exhibit impaired TNF-α production in response to LPS and bacterial lipopeptides^12^. Hence, we analysed CD300 molecules in response to LPS on monocytes from CBMCs. Our results showed that, opposite to adult monocytes, CBMCs monocytes did not upregulate CD300c cell surface expression and CD300e up-regulation was significantly lower when compared with adult monocytes (Fig. 4b). On the other hand, CD300a and CD300f expression were unaltered after LPS treatment on cord blood monocytes, while it was down-regulated on adult monocytes (Fig. 4b). Altogether, our results showed that LPS differentially regulates CD300 cell surface expression on monocytes from adults and neonates.

### CD300c and CD300e mediated activation is quantitatively different in neonatal monocytes

**Figure 5:**
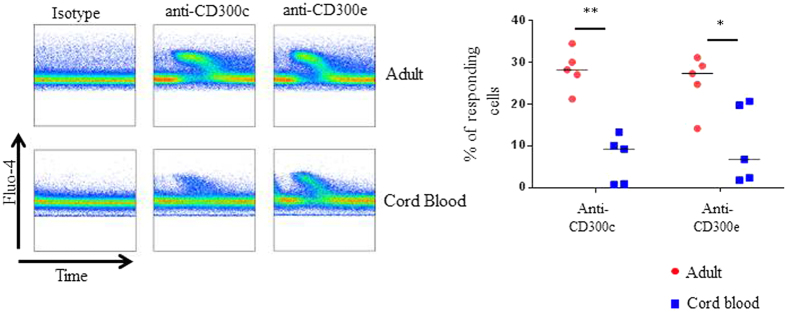
CD300c and CD300e induced calcium mobilization is quantitatively different in neonatal monocytes compared with adult monocytes. Adult PBMCs and CBMCs were loaded with Fluo-4. To establish a baseline, electronically gated monocytes were first acquired for 30 s at which point the primary antibodies, anti-CD300c, anti-CD300e or isotype-matched control, were added. Then at 60 s, the primary antibodies were cross-linked with goat anti-mouse IgE F(ab′)_2_ and fluorescence was measured. Ca^++^ mobilization is represented as an elevation in the fluorescence intensity of the Fluo-4. The pseudocolor representation (left) is a representative experiment. The percentages of responding cells in each experiment were represented in the dot plot graph (right). Each dot represents a different donor, and the medians are shown. *p < 0.05, **p < 0.01.

It is known that CD300c and CD300e are functional activating receptors in adult monocytes^25^,^33^,^34^ and we have shown that there are significant differences in the expression of CD300c, and to a minor extent, in CD300e expression, between monocytes from adults and newborns (Fig. 3a). Therefore, we decided to characterize the function of these two activating receptors in neonatal monocytes. We studied if cross-linking of CD300c and CD300e with specific mAbs was able to induce similar or quantitatively different activation signals in monocytes from cord blood. We first investigated their ability to induce intracellular calcium mobilization as an early event in the signalling cascade mediated by these two receptors. As expected, engagement of CD300c and CD300e with soluble anti-CD300c or anti-CD300e mAbs respectively, followed by cross-linking with anti-mouse IgG F(ab′)_2_, induced transient and rapid increase in intracellular calcium in adult monocytes, which was not observed when cells were stimulated with isotype matched control. However, when we carried out the same assay in monocytes from cord blood we observed significantly less CD300c and CD300e mediated calcium mobilization, indicating that early activation signals through these activating receptors are dampened in neonatal monocytes (Fig. 5).

**Figure 6:**
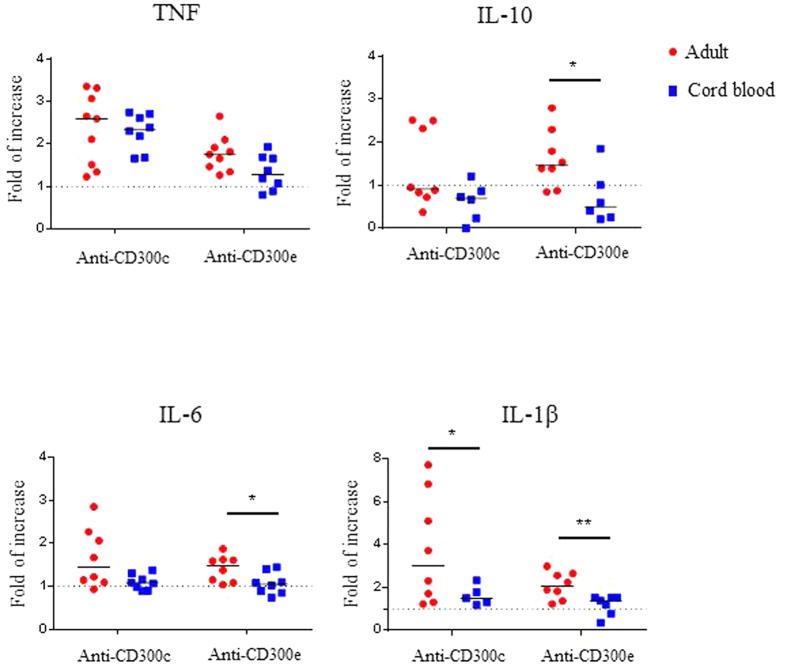
Differential cytokine production after the engagement of CD300c and CD300e in adult and cord blood classical monocytes. Enriched classical monocytes from healthy adult and cord blood were either stimulated with plate bound isotype-matched control antibody, anti-CD300c mAb or anti-CD300e mAb in presence of LPS for 18 h. Culture supernatants were harvested and tested for the secretion of human inflammatory cytokines using flow-cytometric bead analysis. The values on the y-axis correspond to fold of increase over the isotype-matched control antibody for the production of cytokines TNF-α, IL-10, IL-6 and IL1-β. Each dot represents a different donor, and the medians are represented. *p < 0.05, ** p < 0.01.

Then, we determined the ability of CD300c and CD300e to modulate costimulatory molecules’ cell surface expression and cytokine production. In spite of significant differences in calcium mobilization, we only observed a non-significant statistical tendency of neonatal monocytes to exhibit a smaller increase on CD86 expression after CD300c engagement with specific mAbs when compared with adult monocytes (data not shown). We and others have before demonstrated that engagement of CD300c upregulates cytokine secretion from LPS treated monocytes and cross-linking of CD300e induces TNF-α secretion by adult monocytes^25^,^33^. Therefore, we wanted to investigate the role of these two CD300 molecules in the production of cytokines by neonatal monocytes. Freshly isolated classical adult and cord blood monocytes were cultured either with plate-bound anti-CD300c mAb, with anti-CD300e mAb or with isotype-matched control. In contrast to the results obtained with adult monocytes, engagement of CD300c and/or CD300e on neonatal monocytes did not increase LPS-mediated cytokine production, except TNF-α secretion after the cross-linking of CD300c (Fig. 6 and Supplementary Figure 3). Altogether, our results indicate that CD300c and CD300e mediated monocyte activation is significantly reduced in neonatal monocytes, suggesting that it may play a role in the differences between newborn and adult immune responses.

## Discussion

The main objective of this study was to carry out a comparative analysis of the expression of CD300 receptors in neonatal and adult immune cells, along with their regulation and function in monocytes that could help to explain the immaturity of the neonatal immune system. Previous studies have demonstrated that CD300 molecules have a very important role in the responses during viral infections, sepsis, cancer, allergies, autoimmunity and inflammation^36^,^37^,^38^,^39^,^40^,^41^,^42^,^43^,^44^,^45^,^46^,^47^,^48^,^49^,^50^, indicating that this family of receptors is very important for regulating the immune response. Except for a study describing the expression of CD300f on neonatal monocytes^14^, ours is the first comprehensive work examining the expression of CD300 cell surface receptors on human adult PBMCs and CBMCs. Our results show that cells from the neonatal adaptive immune system express low levels of the CD300a inhibitory receptor when compared with adult T and B cells. Specifically, immature B cells and the minor subset of resting memory B cells in cord blood are mostly CD300a negative. Related to T cells, both CD4^+^ T cells and memory CD8^+^ T cells from cord blood exhibited significant lower levels of CD300a when compared with adult T cells. Also, CD300 receptors expression on monocytes from cord blood displayed significant differences when compared with adult monocytes. Furthermore, we also show that LPS differentially regulated CD300 receptors expression on monocytes from adults and neonates, and that CD300c and CD300e mediated activation is quantitatively different in neonatal monocytes compared with adult monocytes.

First, we systematically studied the expression of CD300a, CD300c, CD300e and CD300f on human adult PBMCs. It has been described that transcripts encoding CD300a were present in myeloid and lymphoid cells. Nevertheless, the cell surface expression of CD300a was somewhat difficult to determine due to a lack of specific fluorochrome-conjugated mAbs able to distinguish between CD300a and CD300c. By the simultaneous usage of two fluorochrome-conjugated mAbs that help to differentiate between CD300a and CD300c^25^, we can conclude that human blood T cells only express the CD300a inhibitory receptor on their cell surface. B cells, except for less than 5% of CD300c^+^ memory and TLM B cells, almost exclusively express the CD300a inhibitory receptor. It is important to point out that resting memory B cells conforms a very small subset in cord blood, and the differences between adult and neonates in the expression of CD300 molecules could be due to a different number of both switched and unswitched resting memory B cells on adult and cord blood. More studies are required to address this possibility. On the other hand, CD56^dim^ NK cells express only CD300a, while CD56^bright^ NK cells express CD300a and very low or negative levels of CD300c. We have recently demonstrated that activation with IL-2 and IL-15 of CD56^bright^ adult NK cells, but not CD56^dim^ NK cells, induces the expression of CD300c, while the levels of CD300a were unchanged^51^. Hence, we can conclude that freshly isolated human blood lymphocytes only express the CD300a inhibitory receptor, except for a very small population, less than 5%, of memory and TLM B cells and the very low expression levels of CD300c on the surface of CD56^bright^ NK cells. Both CD300e and CD300f are not expressed on lymphocytes.

On the other hand, the circulating myeloid cells that we have analysed in this report express the four CD300 receptors. However, their expression levels significantly vary between the different monocyte and DC subsets. CD300a and CD300e expression is higher on intermediate and non-classical monocytes, while CD300c is higher in classical monocytes. CD1c^+^ mDCs have higher expression levels of the four CD300 receptors when compared with CD141^+^ mDCs, and pDCs only express CD300a. A previous report analysing the effect of an anti-CD300a/c mAb regulating type I IFN and TNF-α secretion by pDCs^52^ can be ascribed to solely CD300a mediated signals, since we have demonstrated that pDCs only express this inhibitory receptor but not the highly homologous CD300c activating receptor. Until now, the described ligands for human CD300 family of receptors, specifically CD300a and CD300c, are phosphatidylserine (PS) and phosphatidylethanolamine (PE)^34^,^51^,^53^,^54^. PS and ceramide have also been described as ligands for mouse CD300f^41^,^55^, while the ligand of CD300e is unknown. The differential expression of the CD300 receptors combined with the lipid binding ability of some of them suggest that, for example, certain cell types could be more susceptible to infection by viruses that use CD300a to enhance infections^36^ and to protect infected cells from immune cell attack^40^.

The circumstances that naïve CD4^+^ T cells in cord blood do not express CD300a and that there are significantly lower frequencies of CD300a^+^ memory CD4^+^ T cells in CBMCs, may indicate that neonate CD4^+^ T cells are exposed to different signals, that are important for the expression of this inhibitory receptor, than adult CD4^+^ T cells. It is well known that the cytokine milieu is different in foetus and newborns compared with that in adults 10,56,57,58. For example, transforming growth factor (TGF)-β1, a cytokine previously demonstrated to inhibit CD300a expression on adult CD4^+^ T cells^28^, may have an important role in regulating the expression of CD300a on cord blood CD4^+^ T cells. In effect, it has been described that cord blood from healthy newborns after normal spontaneous vaginal delivery displayed high levels of TGF-β1^58^ and that the expression of TGF-β1 is increased in fetal lymph nodes compared with that in adults’ lymph nodes^57^. Also, we have previously shown that *in vitro* cultures with CD4^+^ naïve T cells revealed that Th1 polarization in the presence of IL-12 results in the generation of mostly CD300a^+^ cells and the majority of the IFN-γ producing cells in these cultures were CD300a^+ ^28. Furthermore, expression of CD300a on memory CD4^+^ T cells was associated with Th1 IFN-γ producing cells isolated *ex vivo*^28^. The low frequency of CD300a^+^ memory CD4^+^ T cells in the newborn is consistent with the well-known described defective development of Th1 responses in neonates^56^, which partly is a consequence of a decreased IL-12 production by neonatal APCs^5^,^10^. Similarly, the lower CD300a expression levels on neonate CD8^+^ T cells may be due to the same factors that control its expression on CD4^+^ T cells. The differential expression of CD300 molecules on adult versus neonatal B cells could also be a consequence of the different cytokine environment. High expression of TGF-β1 and a T helper 2 (Th2) skewed response in the neonates^5^,^56^ may be responsible for the significantly low CD300a expression on resting memory B cells. In effect, we have previously shown that the Th2 cytokine IL-4 and TGF-β1 act as negative regulators of CD300a expression on memory B cells^59^. Finally, other factors such as the strength of antigen receptor-mediated signals, TLR-mediated signals and other cytokines, etc., may well have an important and significant role in regulating CD300a and CD300c expression on neonatal circulating lymphocytes.

We have also observed significant differences in the expression pattern of CD300 molecules between monocytes from adults and neonates; specifically in the expression of CD300c, which is significantly less expressed on neonatal monocytes. This lower expression could be the consequence of less exposure and less responsiveness of neonate cells to TLR ligands, such as LPS^12^,^35^. Indeed, our results showing that CD300 molecules expression is significantly less modulated by *in vitro* treatment with LPS supports this conclusion, although we cannot exclude other factors involved in CD300 expression independently of TLR expression. For example, it has been shown that plasma from newborns has the ability to diminish LPS mediated TNF-α release by adult monocytes, suggesting either that newborn plasma is deficient in a factor that enhances ligand-induced TLR activation or that it contains an inhibitor^12^. We have also shown that CD300c and CD300e mediated signals, as shown by intracellular calcium mobilization, are significantly down-regulated in neonatal monocytes when compared with adult monocytes. In the instance of CD300c, this could be easily explained by the reduced expression of this receptor on monocytes from neonates. However, CD300e mediated intracellular calcium mobilization was also significantly down-regulated in neonatal monocytes, while CD300e expression was similar, in classical monocytes, or somehow higher, in intermediate and non-classical monocytes, in newborns compared with adults. This indicates that other factors, such as lower levels of adaptor proteins and/or of signalling intermediates, differences in the agonistic effects of the antibodies, among others, could be responsible for the reduced signalling ability of these two activating receptors on neonatal monocytes. Receptor levels are just one component of the effect of this family of receptors on neonate immunity. Clearly, more studies are required to address this issue.

It is well known that the innate immune system provides the first contact between invading microbes and the host’s defence response. Monocytes are recruited to sites of inflammation where they get activated and secrete a variety of cytokines^60^,^61^. Our results show that while the cross-linking of CD300c and CD300e enhanced LPS-mediated cytokine production by adult monocytes, there was a smaller enhancement (TNF-α) or no enhancement (IL-1β, IL-6 and IL-10), by monocytes from CBMCs. These results are not only explained by the previously described distinct neonatal monocyte responses to LPS, but also by the decreased ability of CD300c and CD300e to signal on newborn monocytes as we have shown here. Therefore, we would like to suggest that the diminished LPS-mediated modulation of CD300 molecules coupled with reduced CD300-mediated signals could help to partially explain the differences on monocyte responses between adults and neonates.

We conclude that neonatal innate and adaptive immune cells exhibit a distinctive pattern of CD300 receptor family expression from adults. We also demonstrate that CD300c and CD300e-mediated activation signals are quantitatively different between adult blood and cord blood monocytes. Therefore, this study adds to our understanding about the function of the neonatal immune system.

## Methods

### Cell isolation and enrichment

Adult and cord blood samples from healthy donors were collected through the Basque Biobank (http://www.biobancovasco.org) under an institutional review board-approved protocol by the Basque Committee of Ethics and Clinical Research. The methods were carried out in accordance with the approved guidelines. The Basque Biobank complies with the quality management, traceability and biosecurity, set out in the Spanish Law 14/2007 of Biomedical Research and in the Royal Decree 1716/2011. All study subjects provided written informed consent. Adult PBMCs and CBMCs from cord blood were obtained by ficoll density centrifugation. Monocyte enrichment was carried out by a negative selection method using the Human Monocyte Isolation Kit II from Miltenyi Biotec.

### Flow cytometry analysis

The following fluorochrome conjugated anti-human monoclonal antibodies (mAbs) were used for the flow-cytometric analysis: PE-Cy7 anti-CD3 (clone SK7), BV421 anti-CD4 (clone RPA-T4), FITC anti-CD8 (clone RPA-T8), PerCP-Cy5.5 anti-CD10 (clone HI10a), PE-Cy7 anti-CD14 (clone MφP9), BV510 anti-CD19 (clone SJ25C1), PE-Cy7 anti-CD20 (clone 2H7), BV510 anti-CD45RA (clone HI100), BV421 anti-CD66b (clone G10F5), BV510 anti-CD141 (clone 1A4), PerCP-Cy5.5 anti-HLA-DR (clone G46-6) and FITC Lineage cocktail 3 from BD Biosciences; FITC anti-CD21 (clone BL13) and PE anti-CD300a-c (clone E59.126) from Beckman-Coulter; PE-Cy7 anti-CD1c (clone L161), FITC anti-CD16 (clone B73.1) and APC anti-CD56 (clone MEM-188) from BioLegend; APC anti-CD300e (clone UP-H2) and PE anti-CD300f (clone UP-D2) from Miltenyi Biotec; APC-eFluor780 anti-CD27 (clone O323), PE and eFluor660 anti-CD300c (clone TX45) and PerCP-eFluor710 anti-HLA-DR (clone L243) from eBioscience. Adult PBMCs, CBMCs and enriched monocytes were washed with staining buffer containing 1X phosphate-buffered saline (PBS) and 1% of fetal bovine serum (FBS) to block Fc receptors and stained with the respective fluorochrome conjugated antibodies for 30 min on ice. Then, cells were washed to remove unbound antibodies and further acquired in a FACSCanto II Flow cytometer (BD Biosciences). We used fluorescence minus one (FMO) control, which contains all the fluorochrome conjugated mAbs in a panel, except for the one that is being measured. FMO control is used to identify and gate cells in the context of data spread due to the multiple fluorochromes in a given panel. Flow cytometry data were analysed by using FlowJo software, version 10.0.7 (TreeStar).

### Stimulation of monocytes with Lipopolysaccharide (LPS)

Adult PBMCs and CBMCs were cultured (2 × 10^6^ cells/ml) in Iscove’s Modified Dulbecco’s Medium (IMDM) supplemented with 10% FBS and 1% GlutaMax and stimulated with 10 ng/mL of LPS (Sigma-Aldrich) in a 24-well plate for 24 h. Then, cells were harvested and analysed for CD300 receptors surface expression by flow cytometric analysis. Monocytes were electronically gated according to forward and side scatter parameters and by the expression of CD14.

### Calcium mobilization assay

Freshly isolated adult PBMCs and CBMCs were washed and resuspended in Rosswell Park Memorial Institute (RPMI) 1640 Medium containing 10% FBS (5 × 10^6^ cells/ml). Next, cells were labelled with Fluo-4 (2 μg/ml) from Life Technologies, for 30 min at 30 °C protected from light. Then, cells were washed twice and resuspended at 2 × 10^6^ cell/ml. Aliquots of 1 ml were warmed at 37 °C for 5 min, followed by acquisition in a FACSCanto II flow cytometer. To establish a baseline, cells were first acquired for 30 s without stimuli and then they were cross-linked either with 5 μg of anti-CD300c (clone TX45), anti-CD300e (clone UP-H2) or isotype control (clone MOPC-21) for 30 s, followed by the addition of 8.5 μg of goat anti-mouse IgG F(ab)_2_. Cells were further acquired for 6 more min. Monocytes were electronically gated based on their forward and side scatters properties. The percentage of responding cells was determined by electronically gating monocytes that had higher fluorescence intensity than the baseline between 60 s (after cross-linking of receptors) and 300 s, once cytoplasmic Ca^++^ has reached the basal levels. Data were analysed by using FlowJo software (Treestar).

### Cross-Linking of CD300c and CD300e on monocytes and measurement of cytokine production

Culture plates (48 wells) were coated with 2.5 μg of either anti-human CD300c (clone TX45), anti-human CD300e (clone UP-H2), or isotype control (clone MOPC-21) for 2–3 h at 37 °C. Enriched monocytes from adult PBMCs and CBMCs were then added to the antibody-coated plates (1 × 10^6^ cell/ml) in IMDM medium supplemented with 10% FBS. Monocytes were also stimulated with 1 ng/ml of LPS. After 18 h of incubation, supernatants were harvested and stored at −80 °C. Human Inflammatory Cytokine CBA kit (BD Biosciences) was used for the measurement of cytokine production, following the manufacturer’s protocol. Samples were acquired on FACSCanto II Flow cytometer and analysed with BD FCAP array software (BD Biosciences).

### Statistical Analysis and graphical representation

Data were analysed using GraphPad Prism software. The data were plotted as individual dot graphs showing medians and unpaired nonparametric Mann-Whitney U test was applied.

## Additional Information

**How to cite this article**: Zenarruzabeitia, O. *et al.* The expression and function of human CD300 receptors on blood circulating mononuclear cells are distinct in neonates and adults. *Sci. Rep.* **6**, 32693; doi: 10.1038/srep32693 (2016).

## Supplementary Material

Supplementary Information
