# Supplementary material for: The expression and function of human CD300 receptors on blood circulating mononuclear cells are distinct in neonates and adults
Source: Sci Rep. 2016 Sep 6;6:32693. doi: 10.1038/srep32693 (PMC5011699; doi:10.1038/srep32693)
Supplement: Supplementary Information [file srep32693-s1.pdf]

# Supplementary Information

# The expression and function of human CD300 receptors on human blood circulating mononuclear cells are distinct in neonates and adults.

Olatz Zenarruzabeitia[1], Joana Vitallé[1], Susana García-Obregón[2], Itziar Astigarraga[2,3,4], Cristina Eguizabal[5], Silvia Santos[5], Venkateswara R. Simhadri[6], Francisco Borrego[1,7].

[1]Immunopathology Group, BioCruces Health Research Institute, Barakaldo 48903, Spain.
[2]Pediatric Oncology Group, BioCruces Health Research Institute, Barakaldo 48903, Spain.
[3]Pediatrics Service, Cruces University Hospital, 48903 Barakaldo, Spain.
[4]Department of Pediatrics, Faculty of Medicine and Dentistry. University of the Basque Country, 48940 Leioa, Spain.
[5]Basque Center for Transfusion and Human Tissues, Galdakao 48960, Spain.
[6]Division of Biotechnology Review and Research-I, Office of Biotechnology Products Review and Research, Center for Drug Evaluation and Research, U.S. Food and Drug Administration, Silver Spring, MD 20993, USA.
[7]Ikerbasque, Basque Foundation for Science, Bilbao 48903, Spain.

Address correspondence and reprint requests to Dr. Francisco Borrego, Immunopathology Group, BioCruces Health Research Institute, Plaza de Cruces s/n, Barakaldo 48903, Spain. E-mail addresses: francisco.borregorabasco@osakidetza.eus or pacoborregorabasco@gmail.com

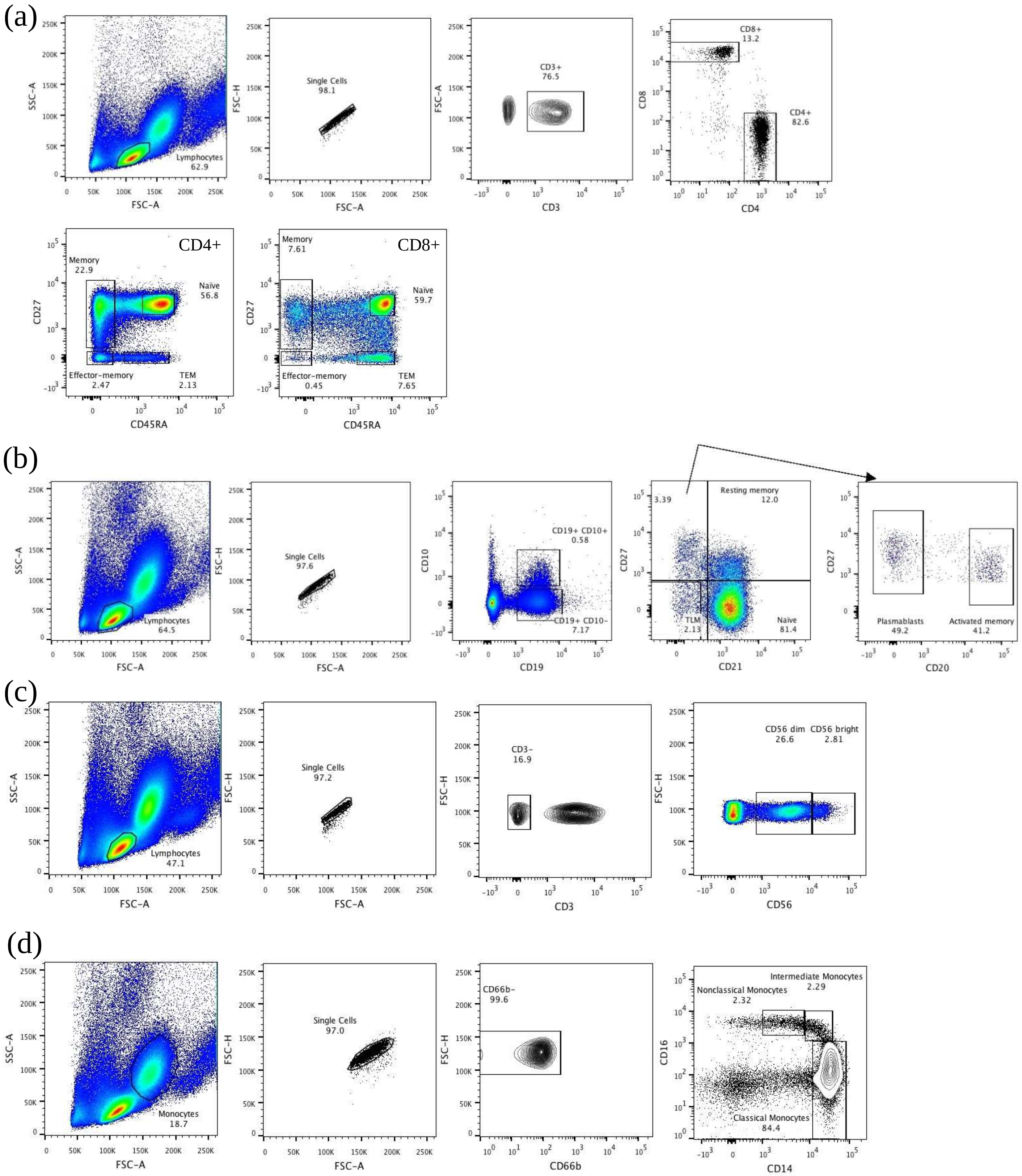
(a)
SSC-A
FSC-A
Lymphocytes
62.9
FSC-H
Single Cells
98.1
CD3+
76.5
CD3
CD8+
13.2
CD4+
82.6
CD8
CD4
CD4+
Memory
22.9
Naive
56.8
Effector-memory
2.47
TEM
2.13
CD27
CD45RA
CD8+
Memory
7.61
Naive
59.7
Effector-memory
0.45
TEM
7.65
(b)
Lymphocytes
64.5
Single Cells
97.6
CD19+ CD10+
0.58
CD19+ CD10-
7.17
CD10
CD19
3.39
Resting memory
12.0
TLM
2.13
Naive
81.4
CD21
Plasmablasts
49.2
Activated memory
41.2
CD20
(c)
Lymphocytes
47.1
Single Cells
97.2
CD3-
16.9
CD56 dim
26.6
CD56 bright
2.81
CD56
(d)
Monocytes
18.7
Single Cells
97.0
CD66b-
99.6
CD66b
Nonclassical Monocytes
2.32
Intermediate Monocytes
2.29
Classical Monocytes
84.4
CD16
CD14

(e)

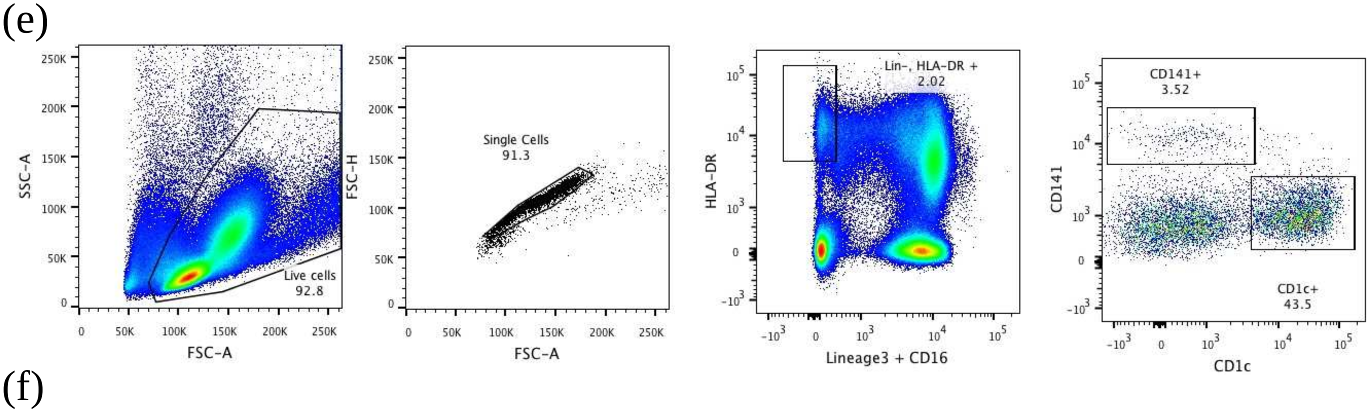


(f)

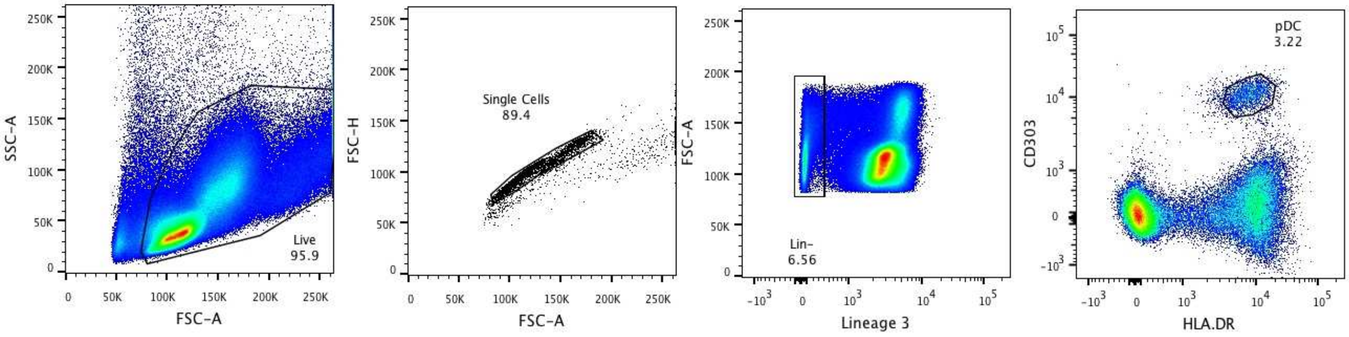


**Supplementary Figure 1.** Gating strategy used for the identification of cell subsets from adult PBMCs and CBMCs. First, cells were electronically gated according to forward and side scatter parameters. Then, single cells were gated according to their area and height. (a) T cells: CD3+ cells were gated and CD4+ and CD8+ subsets were distinguished within the CD3+ T cells. Naïve (CD45RA+CD27+), memory (CD45RA-CD27+), effector/memory (CD45RA-CD27-), and terminal effector/memory (TEM) (CD45RA+CD27-) T cells were distinguished within the CD3+CD4+ and CD3+CD8+ subsets defined by the expression of CD45RA and CD27. (b) B cells: CD19+CD10- (mature) and CD19+CD10+ (immature) B cells were distinguished. Five mature B cell subpopulations were distinguished defined by the expression of CD21, CD27 and CD20: naïve cells (CD21+CD27-), resting memory cells (CD21+CD27+), activated memory cells (CD21-CD27+CD20+), plasmablasts (CD21-CD27+CD20-) and tissue-like memory (TLM) B cells (CD21-CD27-). (c) NK cells: CD3- cells were gated and NK cells were identified as the CD3-CD56dim and CD3-CD56bright populations. (d) Monocytes: CD66b- cells were gated and classical (CD14++CD16-), intermediate (CD14++CD16+) and nonclassical (CD14+CD16+) monocytes were defined by the expression of CD14 and CD16. (e) mDCs: lin-CD16-HLA-DR+ cells were gated, and CD141+ and CD1c+ mDCs were distinguished within them. (f) pDCs: lin- cells were gated, and pDCs were identified within them defined by the expression of HLA-DR and CD303.

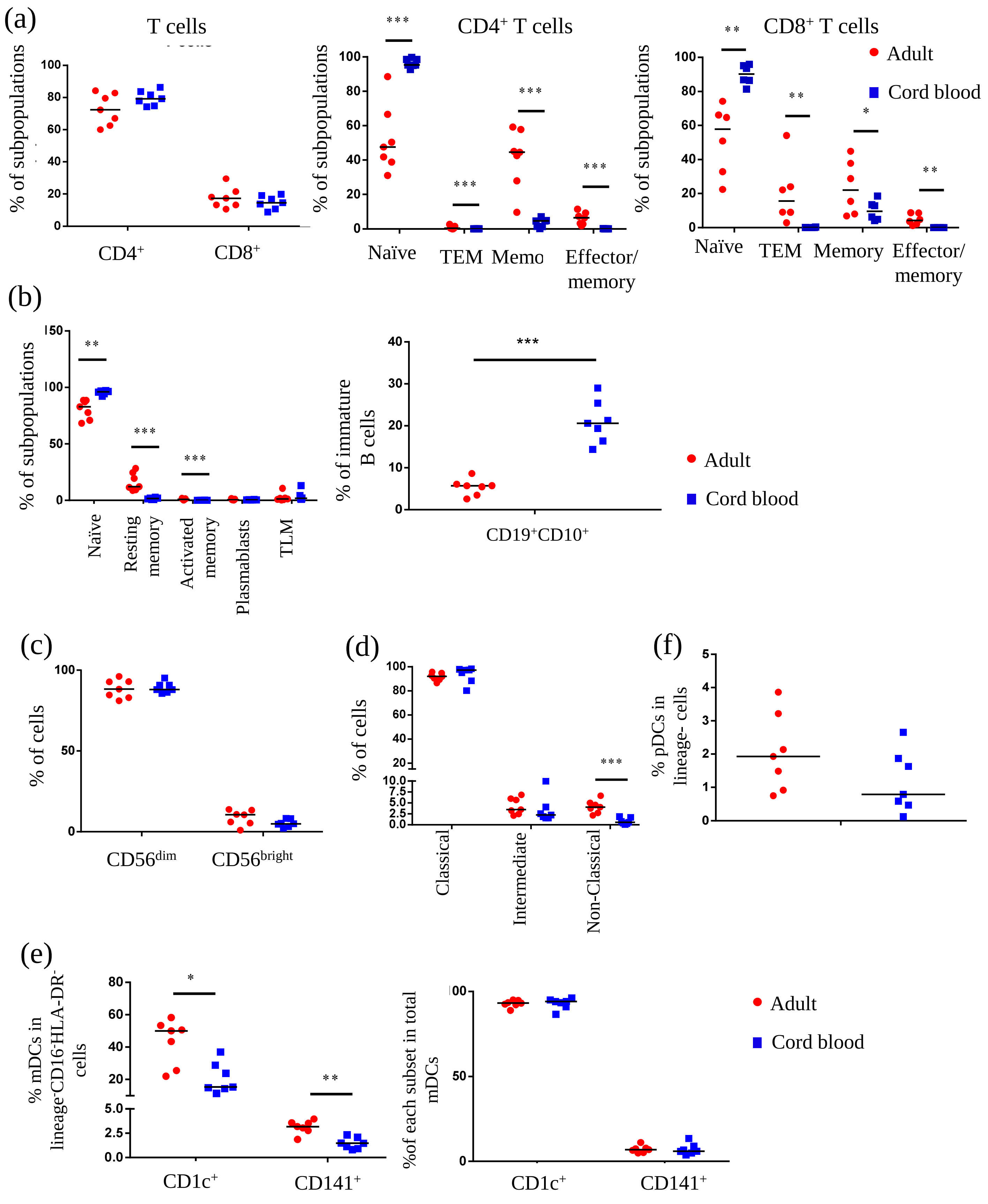


Supplementary Figure 2

**Supplementary Figure 2.** Adult and cord blood immune cell populations and subpopulations. The percentages of each cell populations and subpopulations in adult PBMCs and cord blood CBMCs were represented. Each dot represents a different donor, and the medians are represented. Adults are represented in red and cord blood in blue. (a) T lymphocytes. (b) B lymphocytes. (c) NK cells. (d) Monocytes. (e) mDCs. (f) pDCs. *p<0.05, ** p<0.01, ***p<0.001.

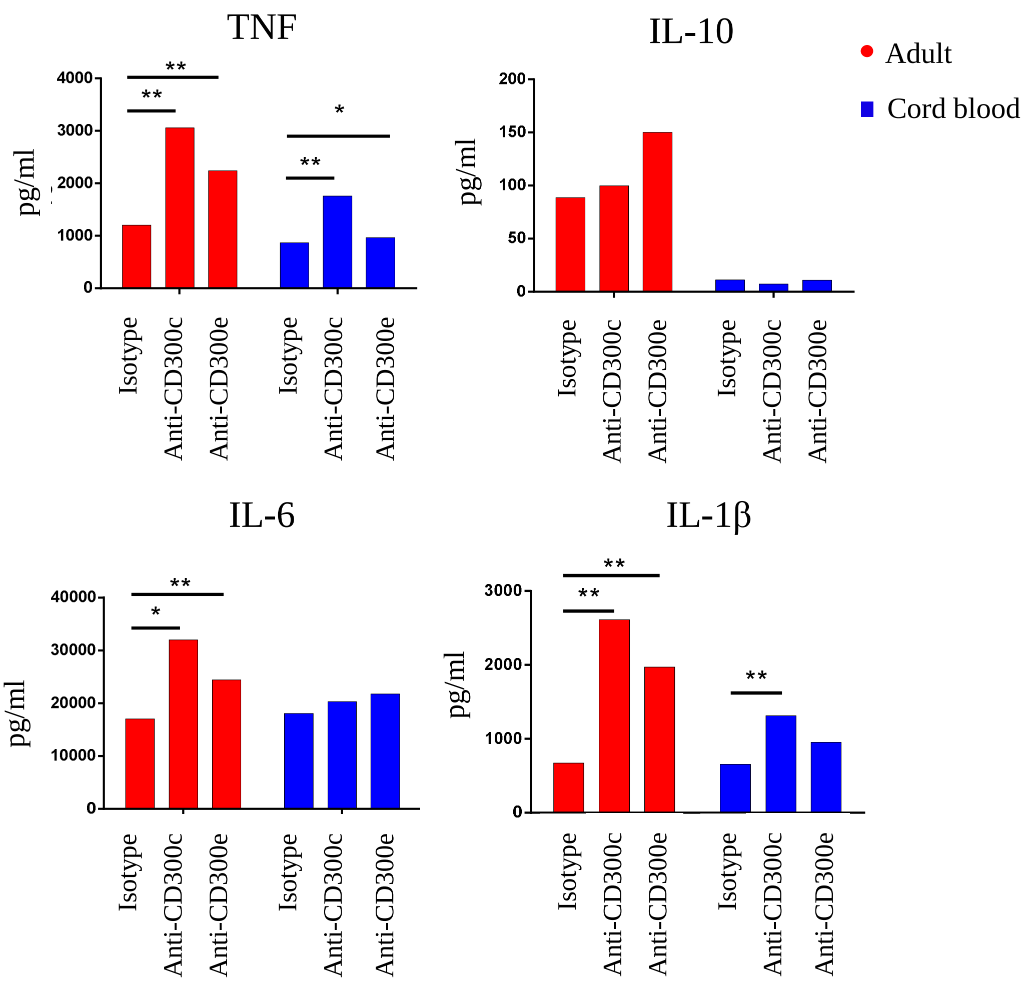


**Supplementary Figure 3. Differential cytokine production after the engagement of CD300c and CD300e in adult and cord blood monocytes.** Enriched monocytes from healthy adult and cord blood were either stimulated with plate bound isotype-matched control antibody, anti-CD300c mAb or anti-CD300e mAb in presence of LPS for 18 h. Culture supernatants were harvested and tested for the secretion of human inflammatory cytokines using flow-cytometric bead analysis. The values on the y-axis correspond to the concentration of cytokines TNF-α, IL-10, IL-6 and IL1-β. Each bar represents the medians. Adults are representing in red and cord blood in blue. *$p<0.05$, ** $p<0.01$.
